# Associations of Tumor Somatic Mutations and Genetic Alterations with Survival Outcomes in Melanoma Patients Treated with Ipilimumab

**DOI:** 10.3390/jcm15062355

**Published:** 2026-03-19

**Authors:** Mohammad Ali Khaksar, Islam Eljilany, Ibrahim Yassine, Xiaoqing Yu, Jamie K. Teer, Jose R. Conejo-Garcia, Maureen Lyons, William LaFramboise, Ahmad A. Tarhini

**Affiliations:** 1 Departments of Cutaneous Oncology and Immunology, H. Lee Moffitt Cancer Center and Research Institute, Tampa, FL 33612, USA; mohammadali.khaksar@moffitt.org (M.A.K.); islam.eljilany@hotmail.com (I.E.); 2 Faculty of Medicine, American University of Beirut, Beirut 1107 2020, Lebanon; iry01@mail.aub.edu; 3 Department of Biostatistics and Bioinformatics, H. Lee Moffitt Cancer Center and Research Institute, Tampa, FL 33612, USA; xiaoqing.yu@moffitt.org (X.Y.); jamie.teer@moffitt.org (J.K.T.); 4 Department of Integrative Immunobiology, Duke Cancer Institute, Duke University School of Medicine, Durham, NC 27710, USA; jose.conejo-garcia@duke.edu; 5 Cancer Genomics Facility, University of Pittsburgh, Pittsburgh, PA 15260, USA; lyonma@upmc.edu; 6 Molecular Diagnostics Laboratory, Allegheny Health Network Cancer Institute, Pittsburgh, PA 15212, USA; william.laframboise@ahn.org

**Keywords:** melanoma, CTLA-4 blockade, ipilimumab, immune checkpoint inhibitor, neoadjuvant, somatic mutation, tumor mutational burden, survival outcomes

## Abstract

**Background:** Identifying patients most likely to benefit from immune checkpoint inhibitors (ICIs) remains a significant challenge in advanced melanoma. We evaluated the association between tumor somatic mutations and clinical outcomes, focusing on relapse-free survival (RFS) and overall survival (OS) in locoregionally advanced melanoma patients treated with neoadjuvant ipilimumab. **Methods:** Tumor specimens and matched peripheral blood samples from 22 patients underwent whole-exome sequencing (WES) to identify non-synonymous somatic mutations. Tumor mutational burden (TMB) was quantified, and specific mutations were analyzed for associations with survival outcomes. **Results:** The analysis revealed a mutational landscape dominated by single-nucleotide missense mutations with a median TMB of 11.4 mutations/MB. *BRAF* and *NRAS* mutations were detected in 73% of patients and exhibited mutual exclusivity and concurrence patterns (*p* < 0.05). Positional clustering identified *NRAS* and *SLC35B4* as key contributors to melanoma (FDR *p*-value < 0.05). Log-rank analysis indicated that mutations in *ODZ1*, *USP34*, *CEP192*, *EML5*, *KIAA1797*, *ATAD5*, and *ANKHD1–EIF4EBP* were associated with shorter survival outcomes (RFS or OS). The associations remained significant in both univariate and multivariable Cox regression models adjusted for TMB. These genes can be broadly grouped into functional categories relevant to tumor progression and immune modulation. In applying multiple testing correction, none maintained statistical significance, indicating that these findings should be interpreted as exploratory and require validation in independent cohorts. **Conclusions:** This study identified tumor genomic alterations associated with clinical outcomes in melanoma patients treated with neoadjuvant ipilimumab, suggesting their potential role in anti-tumor immunity. These findings warrant further investigation in larger cohorts and across other ICIs in melanoma and other malignancies.

## 1. Introduction

The advent of immune checkpoint inhibitors (ICIs) has fundamentally changed the way locoregionally advanced melanoma is treated. By targeting inhibitory immune pathways, including cytotoxic T-lymphocyte-associated antigen-4 (CTLA-4) and programmed death-1 (PD-1), ICIs reinvigorate anti-tumor immune responses, thereby enhancing immune surveillance and promoting tumor regression [1,2]. Despite these advances, clinical responses to ICIs remain highly heterogeneous. While a subset of patients achieves durable clinical benefits, others exhibit limited or no response. Accordingly, there is an obvious need to identify reliable predictive biomarkers that can inform treatment decisions [3].

Accumulating evidence suggests that tumor mutational burden (TMB) and specific somatic mutations may serve as valuable biomarkers of response to ICIs. Elevated TMB, reflecting an increased load of somatic mutations within the tumor genome, has been associated with increased neo-antigen generation and improved immune recognition, thereby augmenting therapeutic efficacy [4]. Additionally, mutations in key oncogenes, including *BRAF* and *NRAS*, influence tumor biology, modulate the immune microenvironment, and contribute to variability in treatment response and resistance, highlighting the value of comprehensive genomic profiling in melanoma management [5,6,7,8]. Large-scale genomic efforts, including those conducted by The Cancer Genome Atlas (TCGA), have significantly advanced understanding of melanoma biology by delineating recurrent driver mutations and their associations with clinical outcomes. However, these datasets are predominantly derived from retrospective analyses or heterogeneous patient populations, which may limit their applicability to specific clinical contexts [9]. In particular, data describing mutational profiles and clinical correlations in the neoadjuvant setting, especially for ipilimumab, a CTLA-4 inhibitor, remain limited. This knowledge gap is particularly significant as neoadjuvant therapy offers an opportunity to evaluate early tumor responses and their potential impact on long-term survival [10].

To address these critical gaps, the present study characterizes the somatic mutational landscape of locoregionally advanced melanoma patients treated with neoadjuvant ipilimumab. Through whole-exome sequencing (WES) of tumor specimens and matched peripheral blood samples, we evaluate associations between tumor-specific genomic alterations and survival outcomes. By identifying genomic features associated with therapeutic benefit, this study provides exploratory insights that could contribute to the development of biomarker-guided stratification approaches in melanoma.

## 2. Materials and Methods

### 2.1. Patient Cohort and Sample Collection

This study (NCT 00972933) included patients diagnosed with locoregionally advanced melanoma who received treatment at the University of Pittsburgh Medical Center (UPMC) previously [11]. Eligibility criteria required availability of tumor tissue and matched peripheral blood mononuclear cell samples (PBMCs) with comprehensive clinical data, including follow-up information regarding disease progression and overall survival. Eligible patients had histologically confirmed locoregionally advanced melanoma and were candidates for neoadjuvant ipilimumab therapy, but had received no prior systemic treatment for advanced disease. The study was approved by the institutional review board at UPMC (IRB# PRO09010033), and all participants in this study (*n* = 22) provided written informed consent [12].

Tumor samples were obtained at baseline prior to initiation of neoadjuvant immunotherapy and again at the time of definitive surgical resection. Specimens were preserved as either snap-frozen tissue or formalin-fixed, paraffin-embedded (FFPE) samples, and tumor tissue domains were macro-dissected to enrich for tumor cell content. Peripheral blood cell samples were collected at the same time points. Genomic DNA was extracted from tissue and blood using the QIAamp DNA Blood Mini Kit (QIAGEN, Hilden, Germany) according to the manufacturer’s instructions. For response assessment, modified WHO criteria were used and imaging studies were performed at baseline (before the initiation of ipilimumab), then at 6–8 weeks after initiation of ipilimumab (before surgery), and subsequently at 3-month intervals. Responses were classified as complete response (CR), partial response (PR), stable disease (SD), or progressive disease (PD). However, radiologic confirmation of responses at 6–8 weeks was not performed due to the planned surgery.

### 2.2. Whole-Exome Sequencing Techniques

Whole-exome sequencing (WES) was performed on all samples using the SOLiD^®^ 5500 Next-Generation Sequencing platform (Thermo Fisher Scientific, Waltham, MA, USA). DNA concentration was measured using a Qubit™ Fluorometer (Thermo Fisher Scientific, Waltham, MA, USA), and DNA purity and quality were assessed by spectrophotometry (NanoDrop ND-1000, Thermo Fisher Scientific) and microcapillary electrophoresis (Bioanalyzer 2100, Agilent Technologies, Santa Clara, CA, USA), respectively. Exome library preparation utilized the SureSelect Human All Exon V5 kit (Agilent Technologies, Santa Clara, CA, USA), capturing approximately 50 Mb of human exonic regions from 5 μg initial DNA substrate. After hybridization to exon-specific probes, the libraries were amplified by polymerase chain reaction (PCR) and paired-end sequencing performed on amplified fragments by sequential oligonucleotide ligation and detection comprising 75 bp reads. Sequencing achieved an average read depth of approximately 100× per exome. Raw sequencing data were processed using SOLiD^®^ BioScope™ software (version 1.3) for base calling and initial quality filtering. Low-quality reads (Phred score < 20) and adapter sequences were removed prior to downstream analysis.

### 2.3. Genomic Analysis

Bioscope software (version 1.3) was used to generate BAM files by aligning filtered color-space sequencing reads to the reference genome (hg19) converted to color space. Duplicate reads were marked using Picard tools, and variant refinement included insertion/deletion realignment and base quality score recalibration using the Genome Analysis Toolkit (GATK) (version 2.2-16). Somatic single-nucleotide variants (SNVs) were identified using MuTect (version 1.1.4) [13] and Strelka (version 1.0.13) [14], and small insertions/deletions (indels) were identified using Somatic Indel Detector (GATK) (part of GATK Lite, version 2.2-16) and Strelka (version 1.0.13). Functional annotation of variants was carried out using ANNOVAR (version 06212012) to classify coding consequences, including missense, nonsense, and splice-site mutations. Tumor mutational burden (TMB) was defined as the total number of protein-altering somatic mutations per exome divided by the number of targeted bases: 61,91,1501 million base pairs (median TMB 11.4, range 1.6–198.4). Positional clustering analysis was performed using OncodriveCLUST to identify mutation hotspots suggestive of driver events. Pathway enrichment analysis was conducted using the maftools package (version 2.22.0) to identify potentially affected biological pathways.

### 2.4. Study Outcomes

Clinical outcomes included relapse-free survival (RFS) and overall survival (OS). Survival time was calculated from the initiation of ipilimumab therapy to the date of the event of interest or the last follow-up.

### 2.5. Statistical Analysis

All statistical analyses were performed using R software (version 4.0.1). Kaplan–Meier survival curves were generated to estimate survival distributions, and differences between groups were compared using the log-rank test. Univariable and multivariable Cox proportional hazards models were utilized to assess associations between somatic mutations across all genes and survival outcomes, including OS and RFS. For each model, hazard ratios (HRs) with 95% confidence intervals (CIs), nominal *p*-values, and Benjamini–Hochberg (BH)-adjusted *p*-values were reported. Multivariable models included TMB as a prespecified covariate based on its established biological relevance in melanoma. To assess sensitivity to model specification, we quantified the absolute change in log hazard ratios (ΔlogHR) following adjustment for TMB. Given the limited cohort size (*n* = 22) and the number of outcome events (7 OS events and 16 RFS events), model complexity was restricted to preserve events-per-variable (EPV) ratios and reduce the risk of overfitting. Univariable models were additionally performed as sensitivity analyses to evaluate the robustness of associations without TMB adjustment. Statistical significance was determined using false discovery rate (FDR)-adjusted *p*-values (<0.05) as the significance threshold. Patterns of mutually exclusive and co-occurring mutations were evaluated using the CoMEt tool. Data visualization was performed using ggplot2 (version 4.0.0) in R (version 4.4.1). Oncoplots of driver mutations were generated using the maftools package (version 2.22.0), and pathway enrichment results were visualized usingmaftools package (version 2.22.0).

## 3. Results

### 3.1. Patient Characteristics and Demographics

This study included 22 patients diagnosed with locoregionally advanced melanoma who were treated with neoadjuvant ipilimumab. Table S1 summarizes the baseline characteristics of the enrolled patients, including age, sex, tumor stage, and Eastern Cooperative Oncology Group (ECOG) performance status, as previously published [12].

### 3.2. Non-Synonymous Mutational Summary

**Figure 1:**
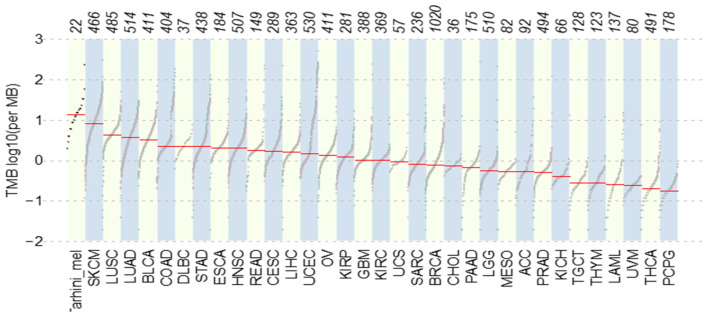
Tumor mutation burden compared with TCGA datasets. The lower X-axis represents the dataset, with the corresponding number of patients in the upper X-axis. The Y-axis denotes the log10 TMB. The red line is the median TMB per dataset. Abbreviations: TMB, tumor mutation burden; TCGA, The Cancer Genome Atlas.

Comprehensive genomic analysis revealed a median TMB of 11.4 (range 1.6–198.4), which is higher than levels reported in TCGA. Marked inter-patient variability in TMB was observed (Figure 1).

### 3.3. Co-Occurrence of Selected Genes

**Figure 2:**
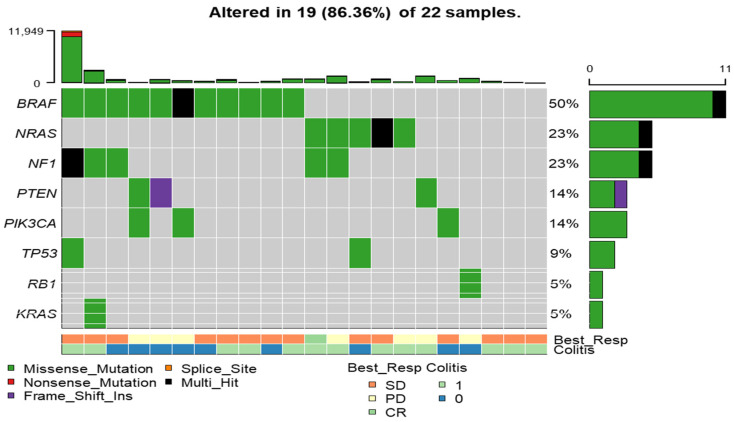
Oncoplot of selected genes. The Y-axis represents a list of eight altered genes in 19 out of 22 patients. The body of the graph displays the type of mutation or alteration, with green indicating missense mutations, black representing multi-hit alterations and violet denoting frameshift mutations. The horizontal bars on the right show the percentage of patients (out of 22) with mutations in each gene. The lower X-axis represents the best response states, categorized as SD (Stable Disease), PD (Progressive Disease), and CR (Complete Response).

As illustrated in Figure 2, alterations in eight genes were identified in 19 of 22 patients (86.36%). Of note, *BRAF* mutations were detected in 50% of patients and were more frequent among those with stable disease (SD; 57%) compared with patients with progressive disease (PD; 43%). However, this difference was not statistically significant (Fisher’s Exact test *p* = 0.66). Additionally, Figure S1 shows the alterations in other genes. Mutations in *NRAS* and *NF1* were detected in 23% of patients, reflecting the heterogeneity of oncogenic drivers in melanoma. Additional alterations were observed in *PTEN* (14%), *PIK3CA* (14%), *TP53* (9%), *RB1* (5%), and *KRAS* (5%), highlighting their roles in cell-cycle regulation and immune evasion. Co-occurrence analysis revealed mutual exclusivity between *BRAF* and *NRAS* mutations (*p* < 0.05), indicating their distinct but overlapping contributions to melanoma oncogenesis. Furthermore, *KRAS* mutations frequently co-occurred with *NF1* alterations, suggesting potential synergistic interactions (Figure S2). Pathway enrichment analysis revealed that RTK-RAS pathway was the most frequently affected oncogenic pathway, followed by NOTCH and WNT pathways (Figure S3). Of note, as shown in Figure S4, positional clustering identified *NRAS* and *SLC35B4* as potential contributors to melanoma.

### 3.4. Association of Mutations with Clinical Outcomes

**Figure 3:**
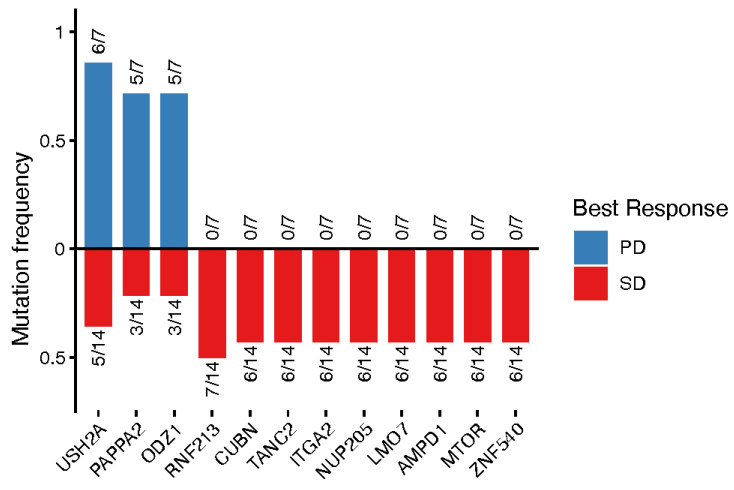
Association of gene mutations and response to therapy. Mutation frequencies of genes significantly associated with therapeutic response (*p* < 0.05, Fisher’s exact test) are shown for patients with progressive disease (PD) and stable disease (SD). Bars represent the fraction of patients harboring mutations in each gene among patients with PD (blue; above the x-axis) or SD (red; below the x-axis). Numbers adjacent to each bar indicate the number of mutated samples relative to the total number of patients in that response group.

As illustrated in Figure 3, mutations in *USH2A*, *PAPPA2*, and *ODZ1* were enriched in patients with PD compared with those with wild-type tumors (*p* < 0.05), with mutation rates of 86%, 74%, and 74%, respectively. In contrast, mutations in the remaining genes were more frequently observed in patients with SD, occurring in approximately 50% of cases.

**Figure 4:**
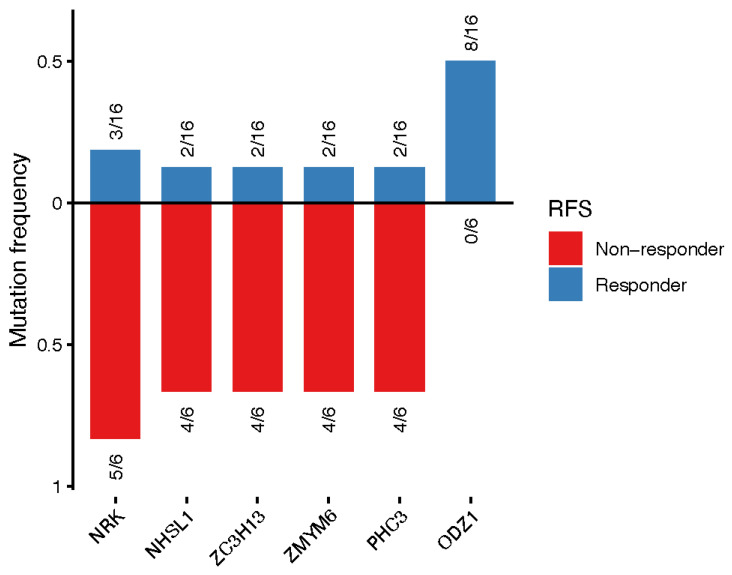
Association of gene mutations and relapse-free survival. Mutation frequencies of genes significantly associated with relapse-free survival (*p* < 0.05, Fisher’s exact test) are shown for responders and non-responders. Bars represent the fraction of patients harboring mutations in each gene within the responder group (blue; above the x-axis) and the non-responder group (red; below the x-axis). Numbers adjacent to each bar indicate the number of mutated samples relative to the total number of patients in the corresponding group.

Genes associated with relapse, as assessed by RFS, are shown in Figure 4. Approximately 66% of patients whose tumors harbored mutations in *NHSL1*, *ZC3H13*, *ZMYM6*, and *PHC3* and 83% of patients whose tumors harbored mutations in *NRK* remained relapse-free compared with those with wild-type alleles. In contrast, 50% of patients with *ODZ1* mutations experienced disease relapse (*p* < 0.05).

**Figure 5:**
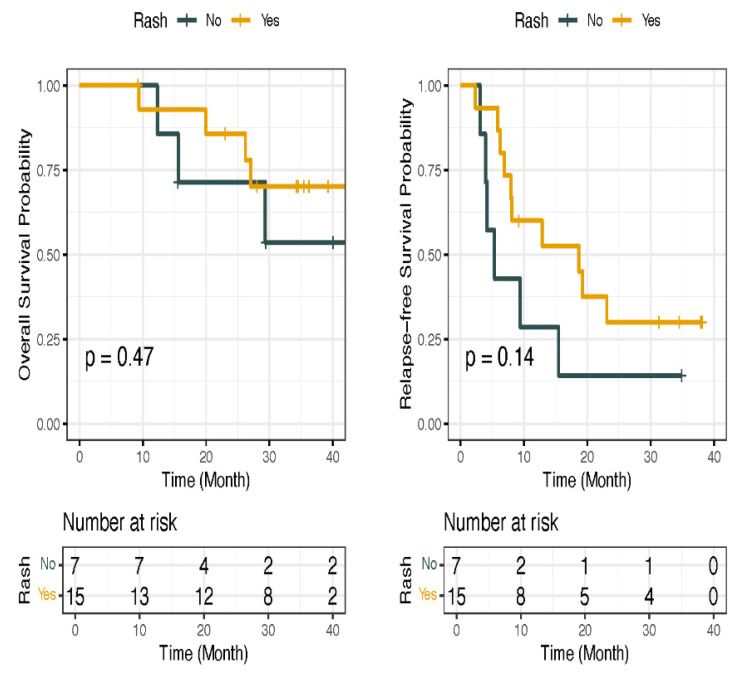
Kaplan–Meier survival analysis stratified by rash status. Kaplan–Meier curves comparing overall survival (OS) (**left**) and relapse-free survival (RFS) (**right**) between patients who developed rash (Yes = 1; orange) and those who did not (No = 0; dark blue). Differences between groups were assessed using the log-rank test. Although patients who developed rash demonstrated numerically longer OS and RFS, these differences did not reach statistical significance (OS: *p* = 0.47; RFS: *p* = 0.14).

**Figure 6:**
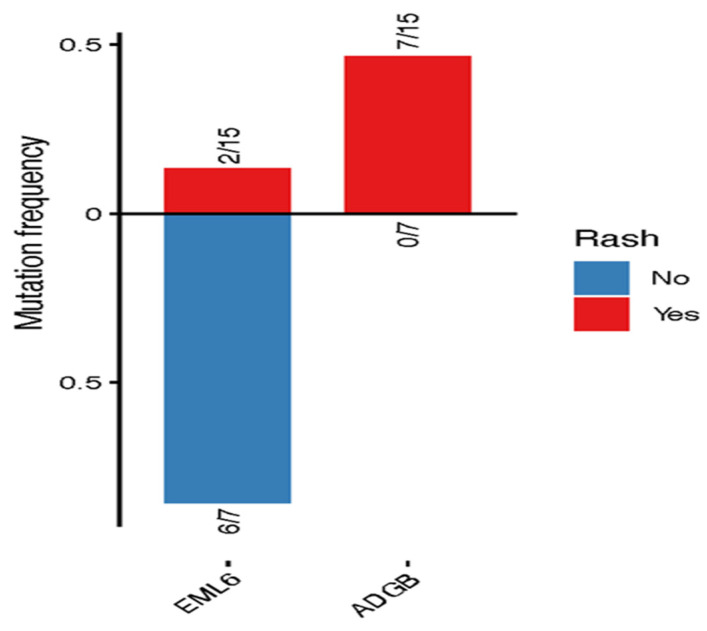
Association between gene mutations and rash development. Somatic mutation frequencies of selected genes in patients who developed rash versus those who did not during immune checkpoint inhibitor (ICI) therapy. Genes shown were significantly associated with rash status (*p* < 0.05, Fisher’s exact test). Bars represent the fraction of patients harboring mutations in each gene within the group without rash (blue; above the x-axis) and the group with rash (red; below the x-axis). Numbers adjacent to each bar indicate the number of mutated samples relative to the total number of patients in the corresponding group.

In the present study (Figure 5), we found that patients who developed rash as an immune-related adverse event (irAE) exhibited longer OS and RFS compared with those who did not develop rash following ipilimumab; however, these differences did not reach statistical significance (OS: *p* = 0.47; RFS: *p* = 0.14). Moreover, *EML6* mutations were more prevalent among patients who did not develop rash and showed no association with survival outcomes (Figure S5), whereas *ADGB* mutations were enriched in patients who developed rash following ICI therapy (Figure 6). Additionally, Kaplan–Meier analyses demonstrated that patients harboring *ADGB* mutations had numerically longer OS and RFS compared with those with wild-type *ADGB*; however, these differences did not reach statistical significance (Figure S6). Furthermore, similar analyses were performed for colitis irAE and the *PTPRO* gene, as described in detail in the Supplementary Results (Figures S7 and S8).

**Figure 7:**
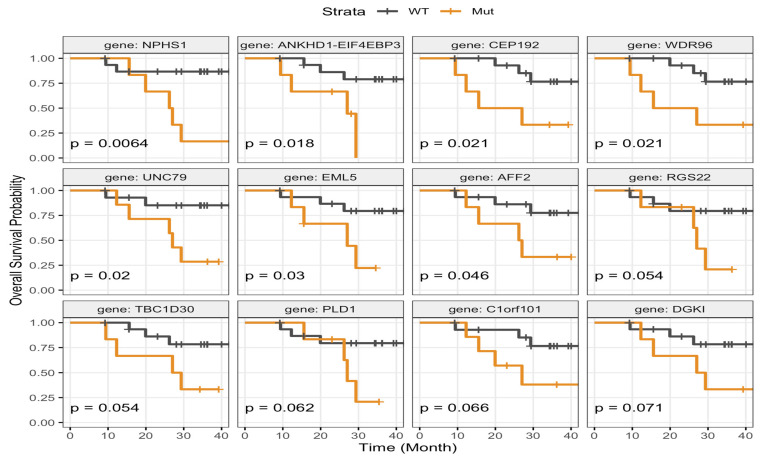
Association of gene-level mutations with overall survival. Kaplan–Meier curves depict OS stratified by mutation status for selected gene. The X-axis represents time (month), and the Y-axis represents overall survival probability. Patients with wild-type tumors are indicated in dark blue, and those harboring mutations are shown in orange.

Using the log-rank test (*p* < 0.05 considered statistically significant), alterations in *NPHS1 (p* = 0.0064), *CEP192* and *WDR96* (both *p* = 0.021), *UNC79* (*p* = 0.02), *EML5* (*p* = 0.03), and *AFF2* (*p* = 0.046) were associated with shorter OS, as demonstrated in Figure 7. Moreover, mutations in *ANKHD1–EIF4EBP3* (*p* = 0.018), a transcriptional readthrough transcript, were also associated with shorter OS. In other words, patients whose tumors harbored the wild-type versions of these genes tended to have longer survival.

**Figure 8:**
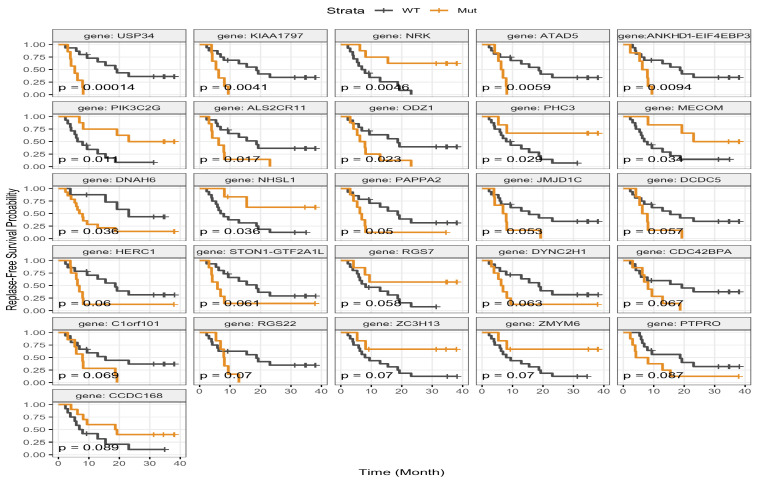
Association of gene-level mutations with relapse-free survival. Kaplan–Meier curves depict RFS stratified by mutation status for selected genes. Time (month) is shown on the X-axis, and RFS probability is shown on Y-axis. Patients with wild-type tumors are indicated in dark blue, and those harboring mutations are shown in orange.

Associations between genomic alterations and RFS are shown in Figure 8. Wild-type alleles of *USP34* (*p* = 0.00014), *KIAA1797* (*p* = 0.0041), *ATAD5* (*p* = 0.0059), *ALS2CR11* (*p* = 0.017), *ODZ1* (*p* = 0.023), and *DNAH6* (*p* = 0.036) were associated with longer RFS. Additionally, mutations in *NRK* (*p* = 0.0046), *PIK3C2G* (*p* = 0.011), *PHC3* (*p* = 0.029), *MECOM* (*p* = 0.034), and *NHSL1* (*p* = 0.036) were also associated with longer RFS.

### 3.5. Cox Proportional Hazard Regression Analysis

Univariable and multivariable Cox proportional hazard regression analyses were performed to evaluate the association between genomic alterations in 339 genes and survival outcomes, including RFS and OS, with tumor mutational burden (TMB) included as covariate (Table S2). As shown in Table 1, univariable Cox regression analysis demonstrated that tumors harboring mutations in *CEP192* (HR = 4.96, 95% CI 1.10–22.37; *p* = 0.03), *WDR96* (HR = 4.96, 95% CI 1.10–22.37; *p* = 0.03), *UNC79* (HR = 5.65, 95% CI 1.09–29.22; *p* = 0.03), *NPHS1* (HR = 7.23, 95% CI 1.38–37.85; *p* = 0.02) and *EML5* (HR = 4.6, 95% CI 1.01–20.85; *p* = 0.04) were associated with shorter OS. After adjustment for TMB in multivariable models, *CEP192* (HR = 5.311, 95% CI 1.03–27.37; *p* = 0.04), *WDR96* (HR = 5.37, 95% CI 1.03– 27.95; *p* = 0.04), *UNC79* (HR = 5.72, 95% CI 1.02–32.07; *p* = 0.04), and *NPHS1* (HR = 7.16, 95% CI 1.31–39.00; *p* = 0.02) retained statistical significance. However, after correction for multiple testing using the false discovery rate (FDR) method, none of these associations remained statistically significant. Similar analyses were performed for RFS, which demonstrated that alterations in *USP34* (univariable: HR = 9.72, 95% CI 2.4–39.3; *p* = 0.001; multivariable: HR = 15.79, 95% CI 3.27–76.32; *p* = 0.0006), *KIAA1797* (univariable: HR = 5.03, 95% CI 1.49–16.98; *p* = 0.009; multivariable: HR = 6.39, 95% CI 1.77–23.06; *p* = 0.004), *ODZ1* (univariable: HR = 3.07, 95% CI 1.11–8.43; *p* = 0.03; multivariable: HR = 3.13, 95% CI 1.13–8.68; *p* = 0.03), *DNAH6* (univariable: HR = 3.25, 95% CI 1.02–10.33; *p* = 0.04; multivariable: HR = 3.40, 95% CI 1.04–11.1; *p* = 0.04)*, ALS2CR11* (univariable: HR = 3.25, 95% CI 1.18–9; *p* = 0.02; multivariable: HR = 3.41, 95% CI 1.21–9.6; *p* = 0.02), and *ATAD5* (univariable: HR = 5.24, 95% CI 1.42–19.33; *p* = 0.01; multivariable: HR = 6.96, 95% CI 1.67–29.04; *p* = 0.007) genes were associated with shorter RFS in both univariate and multivariate Cox regression models. Conversely, patients with tumors harboring mutations in *PIK3C2G* (univariable: HR = 0.23, 95% CI 0.07–0.77; *p* = 0.017; multivariable: HR = 0.15, 95% CI 0.03–0.64; *p* = 0.01), *NRK* (univariable: HR = 0.18, 95% CI 0.05–0.67; *p* = 0.01; multivariable: HR = 0.11, 95% CI 0.02–0.55; *p* = 0.007), *PHC3* (univariable: HR = 0.21, 95% CI 0.05–0.97; *p* = 0.04; multivariable: HR = 0.07, 95% CI 0.006–0.82; *p* = 0.03), *NHSL1* (univariable: HR = 0.23, 95% CI 0.05–1.02; *p* = 0.05; multivariable: HR = 0.093, 95% CI 0.009–0.93; *p* = 0.04), and *MECOM* (univariable: HR = 0.27, 95% CI 0.07–0.98; *p* = 0.04; multivariable: HR = 0.15, 95% CI 0.03–0.81; *p* = 0.03) had improved RFS. However, similar to OS, none of the findings withstood FDR correction. Furthermore, mutations in *ANKHD1–EIF4EBP3*, a transcriptional readthrough transcript, were associated with poorer RFS (univariable: HR = 4.36, 95% CI 1.3–14.58; *p* = 0.01; multivariable: HR = 5.22, 95% CI 1.48–18.46; *p* = 0.01) and OS (univariable: HR = 5.27, 95% CI 1.14–24.4; *p* = 0.03; multivariable: HR = 5.38, 95% CI 1.06–27.43; *p* = 0.04). However, these associations did not remain statistically significant after FDR correction. As a final step, to assess sensitivity to model specification, we quantified the absolute change in log hazard ratios (ΔlogHR) following adjustment for TMB. For most genes, the change in effect size was modest (median |ΔlogHR| = 0.28 for OS and 0.08 for RFS), indicating limited impact of TMB adjustment on the estimated associations (Table S2).

## 4. Discussion

This study identified genomic alterations associated with response to neoadjuvant ipilimumab in locoregionally advanced melanoma, highlighting the complexity of the melanoma genomic landscape and the potential clinical value of comprehensive tumor profiling for risk stratification, outcome prediction, and more tailored immunotherapy approaches in the future. Our findings demonstrated a high prevalence of *BRAF* mutations among our patients, with moderate frequencies of *NRAS* and *NF1* mutations, reflecting the genomic heterogeneity of melanoma. Pathway enrichment analysis demonstrates predominant involvement of the RTK-RAS signaling pathway (Figure S3), reinforcing its central role in melanoma pathogenesis. *BRAF* mutations, particularly the V600E substitution, act as oncogenic drivers by activating the *MAPK* signaling pathway, largely independent of upstream RAS activity, thereby promoting tumor proliferation and disease progression [15]. The higher prevalence of *BRAF* mutations among patients suggests a potentially nuanced interaction between MAPK pathway and partial immune responses to neoadjuvant ipilimumab. While these findings are consistent with the established role of *BRAF* mutations in melanoma biology, they raise questions regarding their association with therapeutic outcomes. While BRAF-targeted therapies have shown efficacy in *BRAF*-mutant melanoma, the enrichment of *BRAF* mutations among patients with stable disease warrants further investigation into their potential influence on responses to ICIs [16,17]. Furthermore, *NRAS* mutations, which activate both MAPK and PI3K pathways, alongside *NF1* mutations that disrupt RAS pathway regulation, underscore the molecular heterogeneity of melanoma and may modulate antitumor immune responses [18,19,20,21,22]. Of note, the prevalence of *BRAF* mutations observed in our cohort is consistent with prior reports in melanoma (approximately 40–60%), while the frequencies of *NRAS* and *NF1* mutations fall within the previously reported range of 15–25% [22,23,24]. Although *BRAF* mutations have been linked to more aggressive disease phenotypes [9,25], a recent study showed that patients with *BRAF* mutant melanoma have consistently demonstrated improved survival compared to those with *BRAF* wild-type tumors [26]. While BRAF-targeted therapies are effective in metastatic disease [27], the relationship between *BRAF* status and ICI responsiveness remains complex and context-dependent, warranting further investigation in larger prospective cohorts.

In this exploratory analysis, several somatic gene alterations were associated with OS and RFS. For instance, alterations in genes such as *CEP192*, *EML5*, *WDR96*, *UNC79*, *NPHS1*, and *ANKHD1–EIF4EBP3* were consistently associated with poorer OS or alterations in genes like *USP34*, *KIAA1797*, *ANKHD1–EIF4EBP3*, *ATAD5,* and *ODZ1* were consistently associated with poorer RFS across analytical approaches. However, interpretation of these associations requires caution. The limited number of OS events (*n* = 7) and RFS events (*n* = 16), combined with a small cohort size (*n* = 22) and multiple testing burden, increases susceptibility to model instability and false discovery. Notably, none of the observed associations retained statistical significance after FDR correction, reinforcing the exploratory nature of these findings. Collectively, these findings may provide preliminary insights into genomic determinants of recurrence risk, but they remain exploratory and require validation in larger cohorts.

To provide biological context, the genes identified in our analysis can be broadly grouped into functional categories relevant to tumor progression and immune modulation. First, alterations in genes involved in centrosome regulation and cytoskeletal organizations such as *CEP192, EML5*, and *ODZ1* were associated with adverse outcomes. *CEP192* is essential for centrosome amplification and has been linked to poor prognosis and immunosuppressive tumor microenvironments in other malignancies [28,29]. *EML5* is involved in regulating microtubule organization and cytoskeletal architecture, which are critical for maintaining cellular integrity and intracellular transport. Disruption of these processes may increase cellular adaptability under therapeutic stress, potentially facilitating tumor survival and disease progression. Although *EML5* is not classified as a canonical oncogenic driver, its dysregulation may be associated with treatment resistance and unfavorable clinical outcomes [30]. With respect to *ODZ1*, a previous study showed an inverse association between the number of ODZ1-positive cells in glioblastoma specimens and both overall survival and progression-free survival. Mechanistically, *ODZ1* enhances tumor aggressiveness by promoting cytoskeletal reorganization through Myc-mediated activation of the RhoA–ROCK signaling pathway, thereby increasing cellular invasion and motility [31]. Second, alterations affecting proliferative and translational signaling pathways were observed, including mutations in *ANKHD1–EIF4EBP3* and *PIK3C2G.*

*ANKHD1* has been identified as a contributor to tumor progression and is considered a potential regulator within the Hippo signaling pathway. In non-small-cell lung cancer, elevated *ANKHD1* expression has been linked to advanced disease stage and poor prognosis, where it enhances cellular proliferation and invasion by activating Yes-associated protein (YAP) and suppressing Hippo pathway activity [32]. *ANKHD1-EIF4EBP3*, also known as *MASK-BP3*–, arises from the alternative splicing and genomic fusion of two functionally distinct loci involved in proliferative signaling and translational control. Given that both *ANKHD1* and *EIF4EBP3* biological roles overlap with Ras/MAPK pathway, this fusion protein may integrate proliferative signaling with translational control, thereby facilitating tumor growth and progression, However, its precise oncogenic role remains to be fully defined [33,34,35,36]. Similarly, Gao et al. [37] reported that loss of *PIK3C2G* expression in A549 cells (a non-small-cell lung cancer cell line) suppressed proliferation, migration, and invasion, while simultaneously promoting apoptosis and disrupting cell cycle progression. Therefore, it is plausible that functionally impairing mutations in *PIK3C2G* may exert anti-tumor effects. Nevertheless, the functional consequences of the specific mutations identified in our cohort require experimental validation.

Third, alterations in genes implicated in genomic stability and tumor suppression, including *ATAD5* and *KIAA1797* (*FOCAD*), were associated with survival outcomes. *ATAD5* regulates proliferating nuclear antigen (PCNA) deubiquitination and maintains DNA replication fidelity. Loss of function in *ATAD5* has been linked to genomic instability and increased susceptibility to several malignancies [38,39,40]. *KIAA1797*, also known as Focadhesin (*FOCAD*), has been implicated in glioblastoma biology. Brockschmidt et al. reported that *KIAA1797* is frequently deleted in glioblastoma [41]. They further demonstrated that restoration of wild-type *KIAA1797* expression significantly reduced colony formation, migration, and invasion in vitro. With respect to *WDR96*, also known as *CFAP43*, and *NPHS1*, which encodes nephrin, a transmembrane protein expressed in kidney podocytes, there are limited studies exploring their roles in malignancies. *WDR96* plays a crucial role in ciliary and flagellar motility, and loss of function alterations in this gene have been linked to male infertility and normal pressure hydrocephalus [42,43]. *NPHS1* mutations account for a substantial proportion of congenital nephrotic syndrome [44].

In addition to the findings mentioned above, Li and Shi reported significant upregulation of *USP34*, a regulator of the Wnt signaling pathway, in acute myeloid leukemia (AML) compared with matched healthy controls and found that elevated expression was associated with adverse clinical outcomes [45,46].

Furthermore, we observed that *ADGB* mutations were associated with the development of rash and improved survival outcomes. Consistent with prior reports, patients who experienced dermatitis irAEs demonstrated prolonged OS and RFS. The development of rash has been proposed as a clinical indicator of enhanced systemic immune activation, potentially reflecting a more robust antitumor immune response. These findings suggest that tumor-intrinsic genomic features may intersect with host immune activation patterns, linking toxicity and therapeutic benefit.

Collectively, our data suggests that in the neoadjuvant setting, somatic alterations beyond canonical driver mutations may contribute to variability in recurrence risk and immune responsiveness. However, the absence of FDR-significant associations, combined with limited event numbers and lack of functional validation, precludes definitive conclusions. These results should therefore be considered hypothesis-generating.

This study has several limitations. The small, single-center cohort limits statistical power and generalizability. The retrospective design precludes causal inference, and intratumoral heterogeneity was not comprehensively assessed. Sampling from a single tumor region may underestimate the genomic complexity influencing therapeutic response. Furthermore, the functional consequences of the identified alterations were not experimentally validated. Despite these limitations, our findings provide preliminary insights into the genomic architecture of melanoma in the neoadjuvant immunotherapy setting and highlight the importance of integrative genomic and clinical analyses. Future studies incorporating larger, multi-institutional cohorts and functional modeling will be essential to validate these associations and clarify the mechanistic interplay between tumor genomics, immune activation, and clinical outcomes.

## 5. Conclusions

The present study identified tumor somatic mutations associated with clinical outcomes in advanced melanoma patients treated with neoadjuvant ipilimumab. Validation in larger cohorts and functional studies are warranted to clarify the biological roles of these genes and their potential contribution to responses to ICIs in melanoma and other malignancies.

## Figures and Tables

**Table 1:** Cox proportional hazards analysis of genomic alterations associated with OS and RFS.

Clinical Outcome	RFS				OS			
Gene	Uni-HR (95%CI)	*p*-Value (FDR)	Mul-HR (95%CI)	*p*-Value (FDR)	Uni-HR (95%CI)	*p*-Value (FDR)	Mul-HR (95%CI)	*p*-Value (FDR)
*USP34*	9.72 (2.40–39.3)	0.001 (0.48)	15.79 (3.27–76.32)	0.0006 (0.2)	2.39 (0.51–11.2)	0.27 (0.99)	2.08 (0.37–11.57)	0.4 (0.93)
*KIAA1797*	5.03 (1.49–16.98)	0.009 (0.97)	6.39 (1.77–23.06)	0.004 (0.6)	2.18 (0.48–9.84)	0.31 (0.99)	1.89 (0.36–10.05)	0.45 (0.93)
*PIK3C2G*	0.23 (0.07–0.77)	0.017 (0.97)	0.15 (0.03–0.64)	0.01 (0.6)	0.17 (0.02–1.47)	0.11 (0.99)	0.001 (0–10,223.668)	0.41 (0.93)
*NRK*	0.18 (0.05–0.67)	0.01 (0.97)	0.11 (0.02–0.55)	0.007 (0.6)	0.18 (0.02–1.55)	0.12 (0.99)	0.002 (0–10,726.719)	0.43 (0.93)
*PHC3*	0.21 (0.05–0.97)	0.04 (0.97)	0.07 (0.006–0.82)	0.03 (0.8)	0.892 (0.17–4.61)	0.89 (0.99)	0.49 (0.05–4.68)	0.53 (0.93)
*ANKHD1-EIF4EBP3*	4.36 (1.3–14.58)	0.01 (0.97)	5.22 (1.48–18.46)	0.01 (0.6)	5.27 (1.14–24.4)	0.03 (0.99)	5.38 (1.06–27.43)	0.04 (0.93)
*NHSL1*	0.23 (0.05–1.02)	0.05 (0.97)	0.093 (0.009–0.93)	0.04 (0.8)	0.4 (0.05–3.3)	0.39 (0.99)	0.002 (0–119,096.794)	0.49 (0.93)
*ODZ1*	3.07 (1.11–8.43)	0.03 (0.97)	3.13 (1.13–8.68)	0.03 (0.8)	3.64 (0.76–17.51)	0.1 (0.99)	3.40 (0.65–17.71)	0.14 (0.93)
*ADGB*	0.44 (0.14–1.39)	0.16 (0.97)	0.32 (0.08–1.29)	0.11 (0.8)	0.28 (0.03–2.3)	0.23 (0.99)	0 (0–8,642,467.025)	0.43 (0.93)
*EML5*	1.9 (0.65–5.54)	0.24 (0.97)	2.05 (0.63–6.62)	0.23 (0.85)	4.6 (1.01–20.85)	0.04 (0.99)	4.67(0.91–23.88)	0.06 (0.93)
*DNAH6*	3.25 (1.02–10.33)	0.04 (0.97)	3.40 (1.04–11.1)	0.04 (0.8)	-	-	-	-
*CEP192*	1.56 (0.54–4.51)	0.41 (0.97)	1.65 (0.49–5.46)	0.41 (0.95)	4.96 (1.10–22.37)	0.03 (0.99)	5.31 (1.03–27.37)	0.04 (0.93)
*BRAF*	1.848 (0.69–5)	0.22 (0.97)	1.9 (0.67–5.3)	0.22 (0.85)	1.53 (0.34–6.9)	0.58 (0.99)	1.31 (0.26–6.52)	0.74 (0.93)
*ALS2CR11*	3.25 (1.18–9)	0.02 (0.97)	3.41 (1.21–9.6)	0.02 (0.8)	0.95 (0.18–4.99)	0.96 (0.99)	0.67 (0.09–4.93)	0.7 (0.93)
*MECOM*	0.27 (0.07–0.98)	0.04 (0.97)	0.15 (0.03–0.81)	0.03 (0.8)	0.29 (0.03–2.48)	0.26 (0.99)	0.002 (0–23,803.775)	0.46 (0.93)
*WDR96*	1.83 (0.63–5.31)	0.26 (0.97)	2.11 (0.62–7.16)	0.23 (0.85)	4.96 (1.10–22.37)	0.03 (0.99)	5.37 (1.03–27.95)	0.04 (0.93)
*NPHS1*	2.33 (0.79–6.82)	0.12 (0.97)	2.38 (0.79–7.15)	0.12 (0.80)	7.24 (1.38–37.85)	0.02 (0.99)	7.16 (1.315–39)	0.02 (0.93)
*UNC79*	1.75 (0.62–4.92)	0.29 (0.97)	1.88 (0.60–5.88)	0.28 (0.90)	5.65 (1.09–29.22)	0.04 (0.99)	5.73 (1.02–32.07)	0.04 (0.93)
*AFF2*	1.656 (0.57–4.83)	0.35 (0.97)	1.66 (0.55–5.04)	0.37 (0.95)	4.13 (0.91–18.66)	0.06 (0.99)	3.96 (0.81–19.31)	0.08 (0.93)
*ATAD5*	5.24 (1.42–19.33)	0.01 (0.97)	6.96 (1.67–29.04)	0.007 (0.60)	1.39 (0.26–7.32)	0.70 (0.99)	0.96 (0.12–7.76)	0.97 (0.99)

HR, Hazard ratio; Uni, univariable; Mul, multivariable; CI, confidence interval; OS, Overall survival; RFS, Relapse-free survival.

## Data Availability

All results relevant to this study are included in the article and Supplementary result. Data is available from the corresponding author upon reasonable request.

## Supplementary Materials

The following supporting information can be downloaded at: https://www.mdpi.com/article/10.3390/jcm15062355/s1, Table S1: Patient demographics and baseline disease characteristics; Figures S1–S8: Additional genomic and survival analyses. Table S2: Results of univariable and multivariable Cox proportional hazards regression analyses for genomic alterations associated with relapse-free survival (RFS) and overall survival (OS), including hazard ratios (HR), 95% confidence intervals (CI), *p*-values, FDR-adjusted *p*-values (q-values), and Δlog(HR).
