# Supplementary material for: Associations of Tumor Somatic Mutations and Genetic Alterations with Survival Outcomes in Melanoma Patients Treated with Ipilimumab
Source: J Clin Med. 2026 Mar 19;15(6):2355. doi: 10.3390/jcm15062355 (PMC13026618; doi:10.3390/jcm15062355)
Supplement: Supplementary file 1 [file jcm-15-02355-s001.zip › Supplementary file. 1.23.2026.pdf]

Supplementary Materials

1. Table S1. Patient demographics and baseline disease characteristics
2. Figure S1. Oncoplot of top mutated genes
3. Figure S2. Pairwise co-occurrence and mutual exclusivity analysis
4. Figure S3. Enrichment of known oncogenic signaling pathways
5. Figure S4. Genomic positional clustering of somatic mutations identifies melanoma driver genes
6. Figure S5. Kaplan–Meier survival analysis according to EML6 mutation status
7. Figure S6. Kaplan–Meier survival analysis according to ADGB mutation status
8. Figure S7. Kaplan–Meier survival analysis stratified by colitis status
9. Figure S8. Kaplan–Meier survival analysis according to PTPRO mutation status

Table S1. **Patient demographics and baseline disease characteristics**

Variable	Patients (N = 27)
Age, years; Median (range)	53 (40–87)
Primary melanoma site, n (%)	
Cutaneous primary	23 (85)
Mucosal primary	3 (11)
Unknown primary	1 (3)
Sex, n (%)	
Female	9 (33)
Male	18 (67)
ECOG performance status, n (%)	
0	19 (70)
1	8 (30)
Recurrent disease after prior surgery, n (%)	21 (78)
Presence of in-transit metastases, n (%)	15 (55)
Estimated risk stage, n (%)	
IIIB	3 (11)
IIIC	24 (89)

Given Figure S1, more than 80% of patients had mutation in *MUC16* gene, followed by *CSMD1* (68%), *PCLO* (68%), and *DNAH4* (64%).

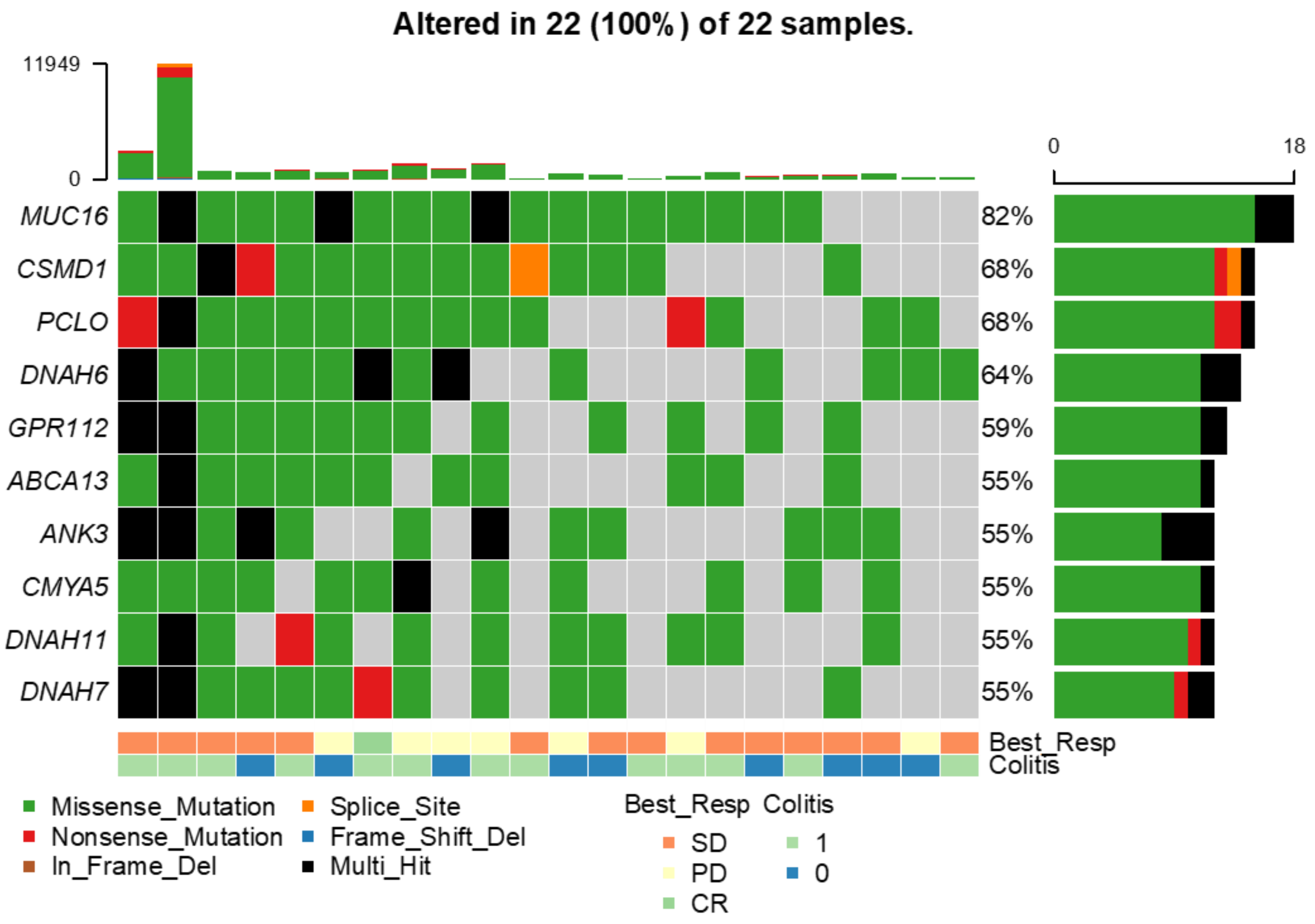

Figure S1. Oncoplot of top mutated genes. The Y-axis represents a list of ten top genes in all 22 patients. The body of the graph displays the type of mutation or alteration, with green indicating missense mutations, black representing multi-hit alterations, red denoting nonsense mutations, and orange demonstrating splice-site mutation. The horizontal bars on the right show the percentage of patients (out of 22) with mutations in each gene. The lower X-axis represents the best response states, categorized as SD (Stable Disease), PD (Progressive Disease), and CR (Complete Response).

Pairwise analysis of the top 25 most frequently mutated genes revealed distinct patterns of mutual exclusivity and co-occurrence across the cohort. Most gene pairs demonstrated mutual exclusivity, indicating that these alterations tend not to co-occur within the same tumors. In contrast, several gene pairs exhibited significant co-occurrence, as indicated by positive associations and statistical significance. These findings highlight non-random patterns of genomic alteration and suggest structured relationships among recurrently mutated genes in this cohort. (Figure S2)

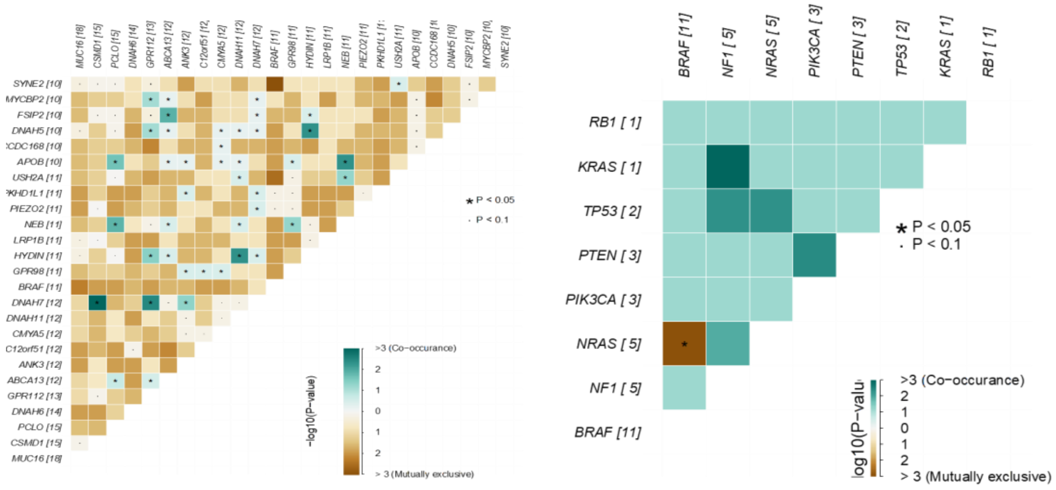

Figure S2. Pairwise co-occurrence and mutual exclusivity analysis. The left-hand Heatmap illustrates pairwise associations among the top 25 most frequently mutated genes across the cohort. Colors indicate the direction and strength of association between gene pairs, with positive values (green/teal) representing co-occurrence and negative values (brown/gold) indicating mutual exclusivity. Near-neutral colors denote weak or no association. Statistical significance of pairwise relationships was assessed using Fisher's exact test, with significant associations denoted by asterisks (*). Right-hand heatmap indicates pairwise associations among selected genes.

As illustrated in Figure S3, pathway enrichment analysis revealed that *RTK-RAS* (58%) was the most affected oncogenic signaling pathway, followed by *NOTCH* and *WNT*.

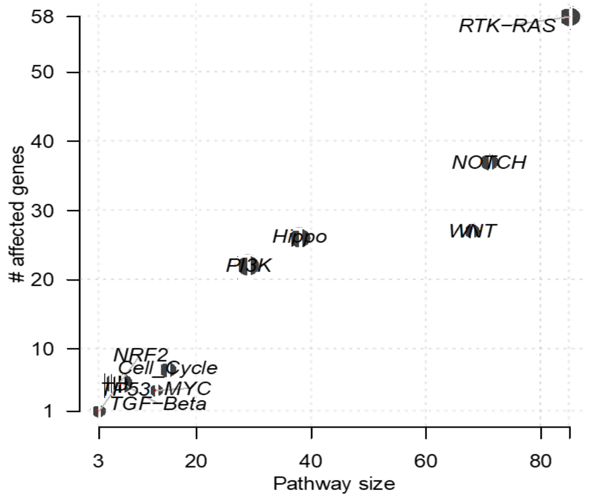

Figure S3. Enrichment of known oncogenic signaling pathways. Enrichment analysis of TCGA-defined oncogenic signaling pathways based on nonsynonymous somatic mutations identified in the cohort. Each point represents an oncogenic pathway, with positioning reflecting the relative degree of pathway alteration. The analysis highlights predominant enrichment of the *RTK–RAS* pathway, followed by *NOTCH* and *WNT* signaling pathways.

As shown in Figure S4, positional clustering identified *NRAS* and *SLC35B4* as key contributors to melanoma (FDR p-value < 0.05).

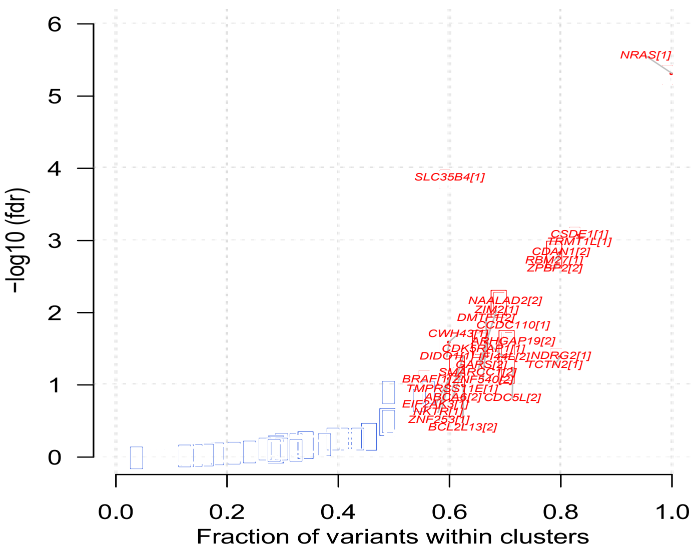

Figure S4. Genomic positional clustering of somatic mutations identifies melanoma driver genes. Genes shown in red (*NRAS* and *SLC35B4*) exhibit significant positional clustering after FDR correction (p < 0.05).

Kaplan–Meier survival analyses showed no significant differences in overall survival (OS) or relapse-free survival (RFS) between patients with *EML6*-mutant tumors and those with wild-type *EML6*. log-rank testing did not demonstrate statistical significance for either OS ($p = 0.84$) or RFS ($p = 0.75$). These findings suggest that *EML6* mutation status was not associated with survival outcomes in this cohort. (Figure S5)

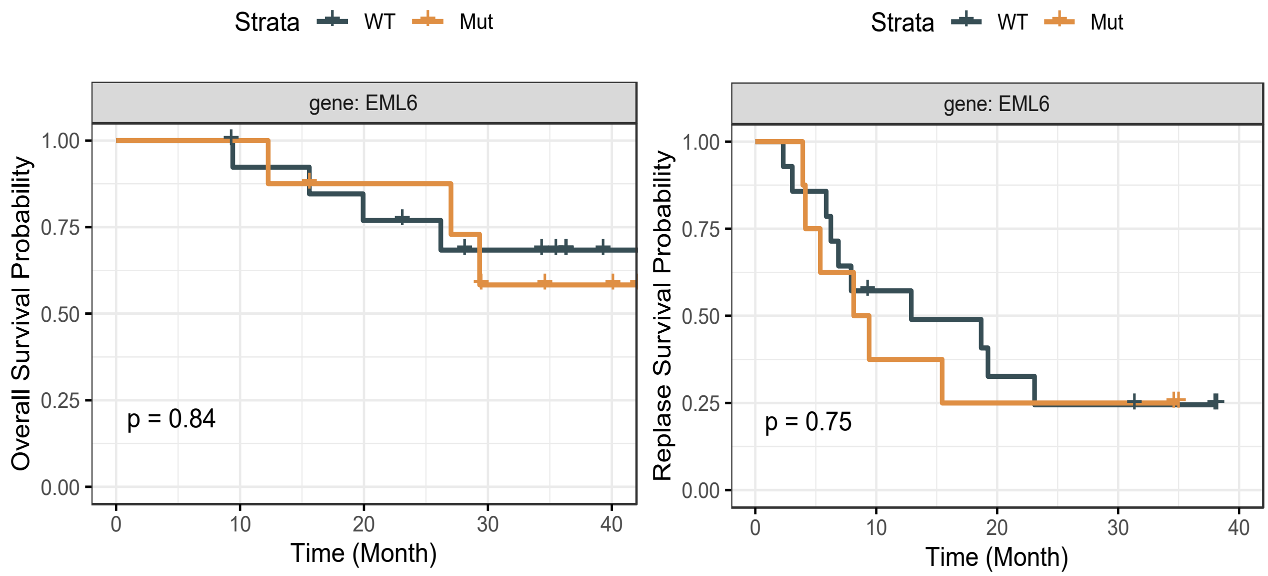

Figure S5. Kaplan–Meier survival analysis according to *EML6* mutation status. Kaplan–Meier curves comparing overall survival (OS) (left) and relapse-free survival (RFS) (right) between patients with *EML6*-mutant tumors (orange) and *EML6*–wild-type (WT) tumors (dark blue). Differences between groups were assessed using the log-rank test. No statistically significant differences in OS or RFS were observed between *EML6*-mutant and wild-type groups (OS: $p = 0.84$; RFS: $p = 0.75$).

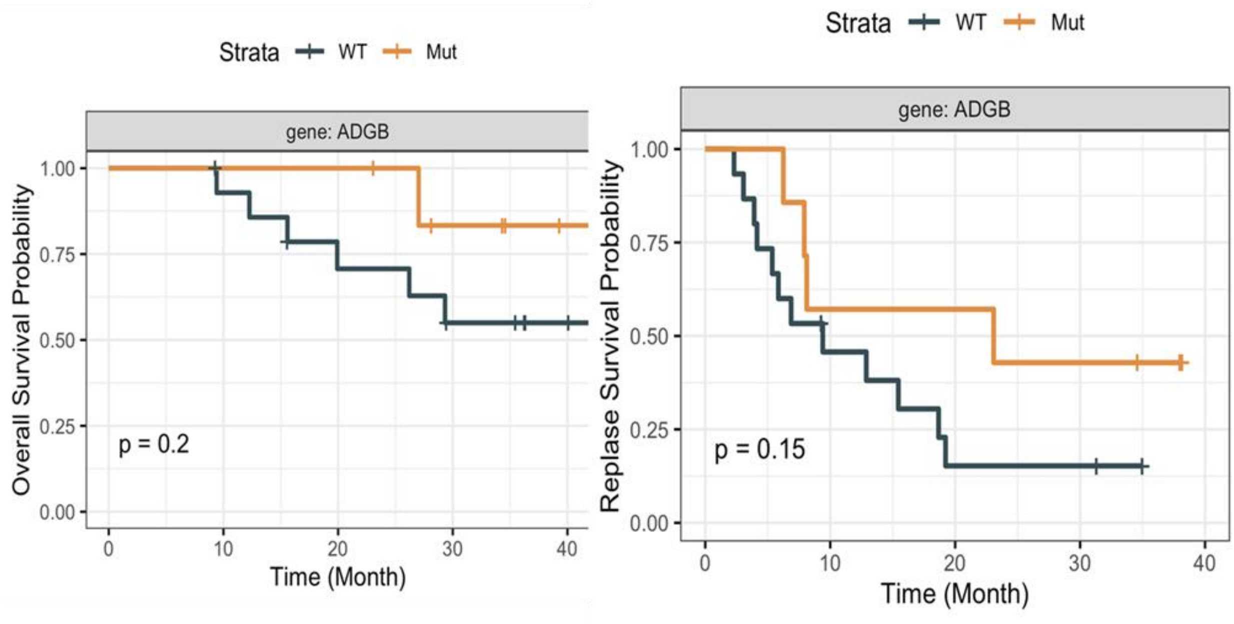

Figure S6. Kaplan–Meier survival analysis according to *ADGB* mutation status. Kaplan–Meier curves comparing overall survival (OS) (left) and relapse-free survival (RFS) (right) between patients with *ADGB*-mutant tumors (orange) and *ADGB*–wild-type (WT) tumors (dark blue). Differences between groups were assessed using the log-rank test. Although patients with *ADGB* mutations demonstrated numerically longer OS and RFS, these differences did not reach statistical significance (OS: $p = 0.20$; RFS: $p = 0.15$)

Kaplan–Meier survival analyses (Figure S7) stratified by colitis status indicated no statistically significant differences in OS or RFS between patients who developed colitis and those who did not during ICI therapy. Although patients who developed colitis showed numerically longer RFS compared with those without colitis, this difference did not reach statistical significance (OS: $p = 0.81$; RFS: $p = 0.084$).

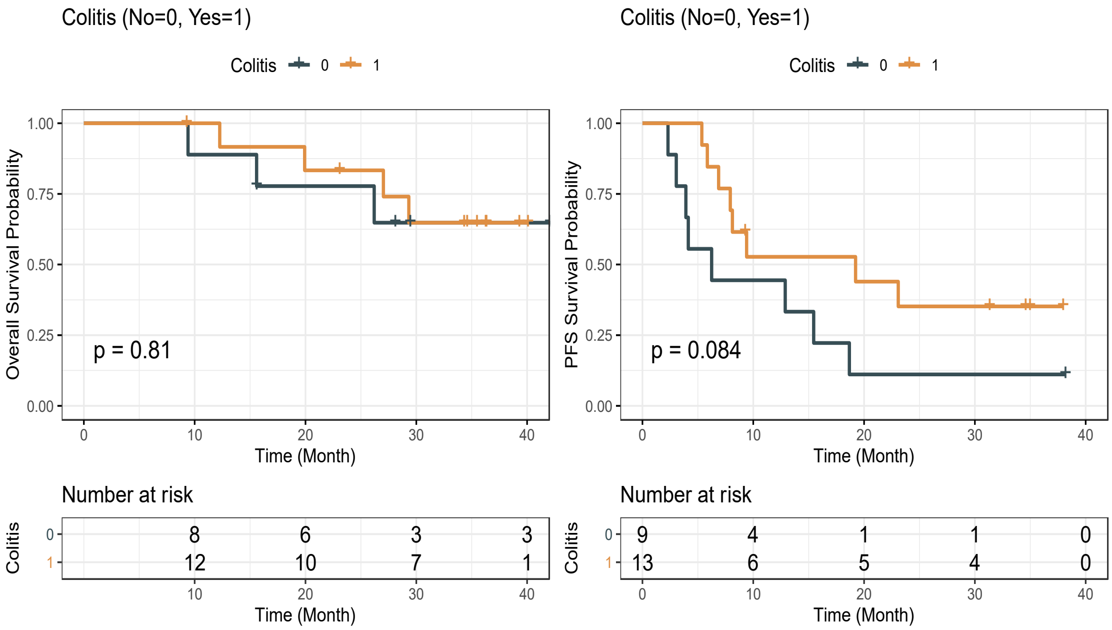

Figure S7. Kaplan–Meier survival analysis stratified by colitis status. Kaplan–Meier curves comparing overall survival (OS) (left) and relapse-free survival (RFS) (right) between patients who developed colitis (Yes = 1; orange) and those who did not (No = 0; dark blue) during ICI therapy. Differences between groups were assessed using the log-rank test. No statistically significant differences in OS or PFS were observed according to colitis status (OS: $p = 0.81$; RFS: $p = 0.084$)

Moreover, given the reported association between *PTPRO* and colitis, we performed Kaplan–Meier survival analyses stratified by *PTPRO* mutation status to assess its potential impact on clinical outcomes. As illustrated in Figure S8, there was no statistically significant differences in OS or RFS were observed between patients with *PTPRO*-mutant and wild-type tumors. Although patients harboring *PTPRO* mutations exhibited numerically shorter OS and RFS compared with those with wild-type *PTPRO*, these differences did not reach statistical significance by log-rank testing (OS: $p = 0.16$; RFS: $p = 0.087$).

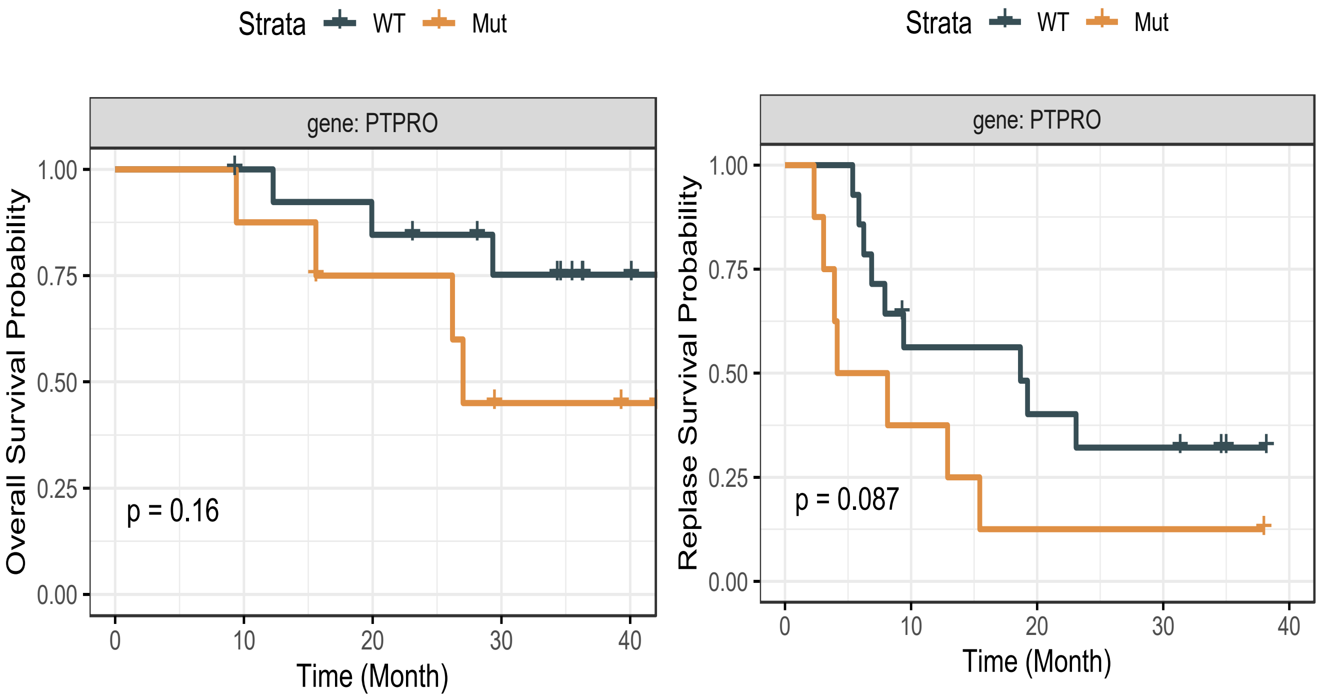

Figure S8. Kaplan–Meier survival analysis according to PTPRO mutation status. Kaplan–Meier curves comparing overall survival (OS) (left) and relapse-free survival (RFS) (right) between patients with PTPRO-mutant tumors (orange) and PTPRO–wild-type (WT) tumors (dark blue). Differences between groups were assessed using the log-rank test. No statistically significant differences in OS or RFS were observed according to PTPRO mutation status (OS: $p = 0.16$; RFS: $p = 0.087$).
